# Effects of a 16-Week Training Program with a Pyramidal Intensity Distribution on Recreational Male Cyclists

**DOI:** 10.3390/sports12010017

**Published:** 2024-01-04

**Authors:** Pedro M. Magalhães, Flávio Cipriano, Jorge E. Morais, José A. Bragada

**Affiliations:** 1 Department of Sports Sciences, Instituto Politécnico de Bragança, 5301-856 Bragança, Portugal; pmaga@ipb.pt (P.M.M.); fcipriano92@gmail.com (F.C.); jbragada@ipb.pt (J.A.B.); 2 Research Center in Sports, Health and Human Development (CIDESD), 6201-001 Covilhã, Portugal

**Keywords:** training effects, somatic characteristics, power at 4 mMol·L^−1^ blood lactate, recreational cyclists

## Abstract

Different training intensity distributions (TIDs) have been proposed to improve cycling performance, especially for high-competition athletes. The objectives of this study were to analyze the effect of a 16-week pyramidal training intensity distribution on somatic and power variables in recreational cyclists and to explore the training zone with the greatest impact on performance improvement. The sample consisted of 14 male recreational cyclists aged 41.00 ± 7.29 years of age. A number of somatic variables were measured. During an incremental protocol, power at a 4 mMol·L^−1^ blood lactate concentration (P4), corresponding power to body mass ratio (P/W P4), and heart rate (HR P4) were also measured. Among the somatic variables, the percentage of fat mass showed the greatest improvement between moments (*p* < 0.001, d = 0.52). Both P4 (*p* < 0.001, d = 1.21) and P/W P4 (*p* < 0.001, d = 1.54) presented a significant increase between moments. The relative improvement in P4 (% P4) showed a significant correlation (R_s_ = 0.661, *p* = 0.038) and relationship (R^2^ = 0.61, *p* = 0.008) mainly with training zone Z2 (blood lactate levels ≥ 2 and <4 mMol·L^−1^). It seems that spending more time in Z2 promoted an improvement in both somatic and power variables in recreational cyclists.

## 1. Introduction

Coaches and amateur cyclists have searched for the best training methods to improve physical performance in cycling, with a limited weekly training volume of 6 to 8 h per week, an average cycling frequency of 2.9 rides/week, and an average weekly distance of 163.9 km for road cyclists [1]. Unlike elite and professional athletes, amateur cyclists normally have full-time jobs outside of cycling. Thus, they typically have less time to devote to training and recovery, and less experience with this type of structured training, so it must be adapted to their reality. Naturally, these athletes will complete a lower total volume of training in their programs, mainly at lower intensities. In fact, professional cyclists ride a total of approximately 30,000 to 35,000 km per year, with a weekly training volume of 700 to 800 km, of which 77 to 78% is at low intensity [2].

A three-zone intensity model is commonly used in the literature to quantify the training intensity distribution (TID), namely based on physiological parameters such as blood lactate levels < 2 mMol·L^−1^ (below lactate threshold 1 (<LaT1) or Zone 1 (Z1)), ≥2 and <4 mMol·L^−1^ (between lactate threshold 1 and 2 (≥LaT1 and <LaT2) or Zone 2 (Z2)), and ≥4 mMol·L^−1^ (above lactate threshold 2 (≥LaT2) or Zone 3 (Z3)), as well as certain percentages of maximal heart rate (HR_max_) or maximal oxygen uptake [3]. The use of a fixed blood lactate threshold during incremental exercise to assess aerobic endurance performance is very common [4], with the LaT2 (4 mMol·L^−1^), originally described by Mader et al. [5], being one of the most widely used [4]. LaT2, or the anaerobic threshold, is a threshold of blood lactate concentration related to an exercise intensity above which the greater recruitment of energy-producing anaerobic pathways is responsible for the onset of fatigue. This threshold has been used as a reference to structure training zones and, in conjunction with session duration, as an indicator of training load [4].

The main types of training intensity distributions used by endurance sports athletes are as follows: (i) pyramidal training; (ii) polarized training; and (iii) threshold training [6]. The literature suggests that polarized training may lead to greater improvements in endurance performance than other intensity distribution approaches [3,7,8]. However, this claim is not supported by all reports, perhaps because of the high and very similar percentage of Z1 in these studies [9,10]; the difference was mainly in volume in Z2 and Z3.

The basic training principles for amateur cyclists are to first ensure consistency, then increase the training load during the training process, balance the intensity distribution during the week/mesocycle/season, and apply the basic principles of periodization and tapering. Typically, recreational and/or regional-level cyclists often have difficulty achieving high volumes in Z1, perhaps because they believe they are not training at an intensity that will promote improvements in their physical condition and performance. There is a common sense that if you do not feel very tired at the end of a training session, you are missing an opportunity to promote adaptations. As a result, these athletes end up spending a lot of time in Z2, where a mixture of energy substrates are used and therefore fat and carbohydrate metabolism are not enhanced in a more significant way. Chronic physiological adaptations to low-intensity training (Z1), particularly those stimulated by calcium-dependent pathways, take time to occur (months or years), but are critical for improving oxidative capacity and lactate clearance, while allowing the body to recover from more intense training sessions [11]. On the other hand, Z2 training can be considered optimal for improving endurance capacity because it provides a significant aerobic training stimulus without the loss of muscle metabolic homeostasis that is characteristic of Z3 [10]. The most intense training zone (Z3) also promotes important physiological adaptations, such as improvements in mitochondrial development and capillary density in type II motor units, possibly through the AMP-activated protein kinase (AMPK) signaling pathway [12]. AMPK is a master regulator that senses the energy state, promotes metabolism for glucose and fatty acid utilization, and mediates beneficial cellular adaptations in many vital tissues and organs. AMPK activation promotes peroxisome proliferator-activated receptor gamma coactivator 1 alpha (PGC-1α) activity, resulting in the translocation of PGC-1α to the nucleus, where it functions to promote the transcription of mitochondrial genes [13].

A relevant physiological adaptation for the improvement in endurance cycling performance is the increase in lactate clearance capacity, mainly by type I muscle fibers, which contributes to the improvement in oxidative capacity. In fact, blood lactate accumulation is negatively correlated with fat oxidation and positively correlated with carbohydrate oxidation during exercise [14]. Therefore, exercise intensities above Z1 raise blood lactate above baseline values, which impairs fat mobilization to skeletal muscle and negatively impacts endurance capacity. Several studies have investigated the effect of different TID types on cycling performance [3,7,8], particularly in well-trained athletes [3,6]. Fewer studies have examined the effect of a 16-week pyramidal TID on the power output at 4 mMol·L^−1^ blood lactate concentrations (P4) in recreational cyclists, and additional experimental studies are needed to clarify the importance of TID in relation to performance [15].

Therefore, the objectives of this study were to analyze the effect of a 16-week pyramidal TID on P4 and power to body mass ratio at P4 (P/W P4) in recreational cyclists and to determine the training zone with the greatest effect on the P4 change. In this sense, it was hypothesized that the proposed TID contributes to the increase in P4 and P/W P4, and that Z1 and Z2 have a significant impact on the improvement in P4.

## 2. Materials and Methods

### 2.1. Research Design and Training Intervention

**Figure 1:**
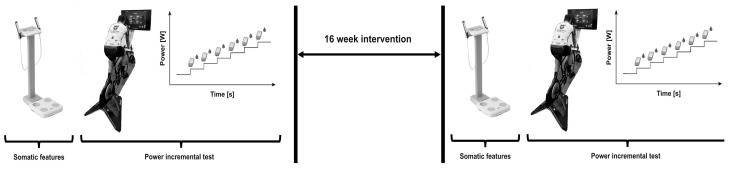
Research design and data collection infographic.

An a priori power analysis was performed using G*Power [16]. Twelve participants were required to detect a large effect size (d = 0.8) with 80% power (α = 0.05, one-tailed test) for a “Means: difference between two dependent means (matched pairs)” statistical test. At the beginning and end of the intervention program, the cyclists underwent a series of measurements. Specifically, these measurements included body composition assessment, determination of power at 4 mMol/L^−1^ of blood lactate (P4), and heart rate during the progressive test (Figure 1).

All subjects were in the early stages of their training process, following a period of infrequent cycling. During the training protocol, all subjects trained three to six times per week (average of 5.21 ± 1.37 sessions per week) for an average volume of 7.57 ± 2.1 h per week for a period of 16 to 18 weeks. The weekly training prescription consisted of one interval session at Z3 (⩒O_2max_), one interval session at Z2 (LaT2), a free session on the weekend, and the remaining training sessions at Z1. The weekend session was usually longer and conducted informally as a group ride. This session was carried out without specific intensity guidelines. Most cyclists had power meters on their bicycles and/or regularly used stationary trainers to control the intensity of their workouts. After each exercise session, the data were downloaded to the Intervals.icu^®^ platform (https://www.intervals.icu accessed on 15 October 2023), where they were subsequently analyzed by the research team, and feedback was provided on the session performed.

The 16-week training program followed a pyramidal training approach, with cyclists advised to devote approximately 60% of their training volume to Z1, 30% to Z2, and the remaining 10% to Z3. Training in Z1 and Z2 was primarily completed during the Sunday training sessions, while Z3 training was completed during interval training sessions.

In cycling, polarized training appears to produce the greatest improvements in key variables of endurance performance (peak oxygen uptake [⩒O_2*peak*_], time to exhaustion [TTE], peak velocity/power [V/Ppeak], and velocity/power at 4 mmol·L^−1^ of blood lactate [V/P4]) when compared to high-intensity interval training (HIIT), high-volume training (HVT), and “threshold training” (THR) [7]. It has also been observed that running a mesocycle of pyramidal periodization, followed by a mesocycle of polarized periodization, seems to lead to more significant adaptations in terms of LaT1, LaT2, absolute and relative ⩒O_2*peak*_, and 5 km running time trial performance, compared to a polarized periodization followed by a pyramidal periodization [17].

Training intensity zones were determined based on typical heart rate (HR) and blood lactate values for these zones (Table 1) [18]. At each assessment point, the power values associated with each zone were determined individually from the data obtained during the progressive test to determine P4.

Table 2 presents data related to training volume. It shows the number of workouts, distance, training stress score (TSS), time, and time per training zone. It also shows the partial contribution of the time spent in each training zone to the total time. Zone 1 (Z1) was defined as blood lactate levels < 2 mMol·L^−1^. Zone 2 (Z2) was defined as blood lactate levels ≥ 2 and <4 mMol·L^−1^. Zone 3 (Z3) was defined as blood lactate levels ≥ 4 mMol·L^−1^ [3]. During different training sessions, cyclists were instructed to monitor training intensity by considering both the power and HR values assigned to each zone in combination, but without exceeding the upper limits values at the same time. During longer training sessions, they were instructed to focus primarily on HR, while during high-intensity or shorter interval sessions, they were instructed to focus on power. The training records of all cyclists were recorded on the free electronic platform Intervals.eu. As these were recreational cyclists, the duration and the number of sessions were not the same for all participants. As a result, the weekly training volume varied. However, the structure of the training plan was similar, including the proportion of training time allocated to each intensity zone.

### 2.2. Participants

The sample consisted of 14 male recreational cyclists who were 41.00 ± 7.29 years old and 1.73 ± 0.05 m tall. Other somatic characteristics can be seen in Table 2, as they were part of the training intervention outcomes. The athletes were all members of a regional cycling club and regularly participated in regional and national competitions (Tier 2 athletes) [19]. Inclusion criteria were (i) over 18 years of age, participating in at least two cycling training sessions per week; (ii) without health limitations and fully fit; and (iii) not taking any regular medication. All procedures were in accordance with the Declaration of Helsinki regarding human research, and the Ethics Board approved the research (No.137/2023).

### 2.3. Data Collection of Somatic Characteristics and Body Composition

Height was measured with a stadiometer (SECA 242, Hamburg, Germany) while the subjects were barefoot. Body mass index (BMI) was calculated from height and body mass. Body mass and body composition variables were measured using a bioimpedance device (Tanita, MC 780-P MA, Tokyo, Japan), according to the manufacturer’s software instructions. Therefore, the evaluation of each subject included the measurement of: (i) body mass (kg); (ii) total body fat and percentage of body fat; (iii) visceral fat level provided by the scale; (iv) muscle body mass and percentage of muscle mass; (v) body mass and percentage of body water. The results of this assessment were provided to the cyclists in a graphical format, with each individual value positioned on a normative chart provided by the GNON Health Monitor software version 3.4.5.

### 2.4. Data Collection of Power at 4 mMol·L^−1^ of Blood Lactate Concentration (P4)

The determination of the power associated with a blood lactate concentration of 4 mMol·L^−1^ (P4) was performed on a TACX ergo trainer (Neo Smart 2T, Wassenaar, The Netherlands) in a progressive, continuous test with four or five levels. This test was preceded by a 10 min warm-up period at a power output of 100 W.

The starting power for the test was determined based on somatic characteristics, years of practice, and some performance data, and started at either 130 W or 150 W. Each stage lasted for 6 min, with increments of 30 W from one stage to the next. Capillary blood lactate concentration was measured immediately after each 6 min stage, without pauses. The Lactate Pro 2 analyzer (Arkray Inc., Kyoto, Japan) was used for this purpose.

The test to determine P4 was terminated when the blood lactate concentration exceeded 4 mMol·L^−1^ at any of the stages performed. Heart rate was measured continuously throughout the test. The average of the last 15 s of each stage was recorded and used as the reference value for that stage.

### 2.5. Statistical Analysis

Normality and homoscedasticity assumptions were analyzed using the Shapiro–Wilk and Levene tests, respectively. Mean, standard deviation (SD), and coefficient of variation (CV = standard deviation/mean × 100, in %) were calculated as descriptive statistics. A paired-samples *t*-test was used to verify the differences between the moments (α = 0.05). The mean difference (MD) with 95% confidence intervals (95CI) was analyzed. Cohen’s d was used to estimate the standardized effect sizes and was considered to be: (i) trivial if 0 ≤ d < 0.20; (ii) small if 0.20 ≤ d < 0.60; (iii) moderate if 0.60 ≤ d < 1.20; (iv) large if 1.20 ≤ d < 2.00; (v) very large if 2.00 ≤ d < 4.00; and (vi) nearly distinct if d ≥ 4.00 [20]. The Spearman correlation coefficient (R_s_) was used to analyze the strength and direction of the correlations between the improvement in P4 with the training zones (α = 0.05). The improvement in P4 was considered as the relative difference (in %) between the pre- and post-test. A simple linear regression was used to understand the relationship between the same pairs. For a qualitative interpretation, the relationship was defined as: very weak if R^2^ < 0.04, weak if 0.04 ≤ R^2^ < 0.16, moderate if 0.16 ≤ R^2^ < 0.49, high if 0.49 ≤ R^2^ < 0.81, and very high if 0.81 ≤ R^2^ < 1.0.

## 3. Results

**Figure 2:**
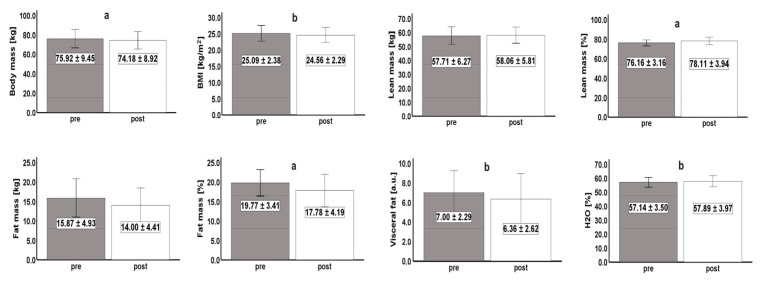
Descriptive data of the somatic variables measured in the pre- and post-tests. BMI—body mass index; H_2_O—water. ^a^—significant difference (<0.001) between pre- and post-test; ^b^—significant difference (<0.05) between pre- and post-test.

Figure 2 shows the descriptive statistics (mean ± SD) of the somatic variables measured in the pre- and post-test. Overall, there was a significant improvement in these characteristics. Body mass (*p* < 0.001), BMI (*p* < 0.05), percentage of fat mass (*p* < 0.001), and visceral fat (*p* < 0.05) decreased significantly between the assessment times. Conversely, lean mass percentage (*p* < 0.001) and water percentage (*p* < 0.05) significantly increased between assessment times (Figure 2).

**Figure 3:**
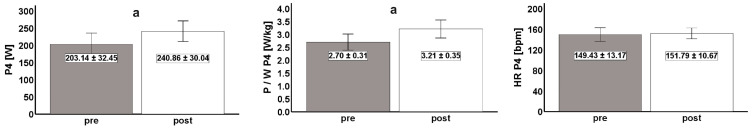
Descriptive data of the power-related variables measured in the pre- and post-tests. P4—power at 4 mMol·L^−1^ of blood lactate concentration; P/W P4—power to body mass ratio at P4; HR P4—heart rate at P4. ^a^—significant difference (<0.001) between pre- and post-test.

Figure 3 shows the variables related to power. These also showed significant improvements between evaluation moments. The power output at 4 mMol·L^−1^ blood lactate concentration (P4) and the power to body mass ratio at P4 (P/W P4) showed significant improvement (*p* < 0.001) over time. The heart rate at P4 (HR P4) did not change significantly over time (Figure 3).

Table 3 presents the data of the paired-samples *t*-test. It is possible to note that the P4 and P/W P4 were the variables with the greatest improvement between assessment moments (P4: d = 1.21, large effect size; P/W P4: d = 1.54, large effect size). This indicates that the training intervention was more likely to elicit the power output for the same blood lactate levels in conjunction with a reduction in the percentage of body fat, resulting in a significant increase in power to body mass ratio. These significant improvements in P4 and P/W P4 are clinically relevant for promoting health and cycling performance and represent a significant improvement in the physical fitness of individuals.

**Figure 4:**
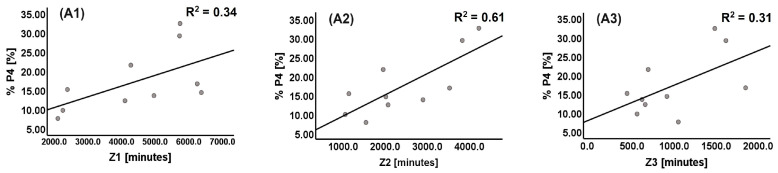
Relationship between P4 improvement (% P4) and each training zone. (**A1**)—relationship between the relative improvement in P4 (% P4) and training zone Z1; (**A2**)—relationship between the relative improvement in P4 (% P4) and training zone Z2; (**A3**)—relationship between the relative improvement in P4 (% P4) and training zone Z3.

The relative improvement in P4 (% P4) showed a significant correlation with Z1 (R_s_ = 0.636, *p* = 0.048) and Z2 (R_s_ = 0.661, *p* = 0.038), but not with Z3 (R_s_ = 0.479, *p* = 0.162). Figure 4 shows the simple linear regression between the improvement in P4 (% P4) with each training zone. The % P4 showed a significant and high relationship with Z2 (R^2^ = 0.61, *p* = 0.008—Panel A2), and moderate but not significant relationships with Z1 (R^2^ = 0.34, *p* = 0.079—Panel A1) and Z3 (R^2^ = 0.31, *p* = 0.093—Panel A3).

## 4. Discussion

### 4.1. Main Findings

The objectives of this study were to analyze the effect of a 16-week pyramidal TID on P4 and P/W P4 in recreational cyclists and to determine the training zone with the greatest effect on P4 improvement. The main results showed that all somatic and power-related variables improved during the 16-week training program. Of the somatic variables, the percentage of fat mass and the percentage of lean mass showed the greatest improvement, with decreases and increases, respectively. Similarly, other studies have shown that both low-intensity training [21] and high-intensity interval training (HIIT) [22,23] significantly contribute to the reduction in fat mass and body fat percentage.

### 4.2. Somatic Findings

In the meta-analysis report by Fatemeh Khodadadi et al. [24], resulting from the analysis of cycling intervention studies with HIIT (training protocols duration between 3 and 15 weeks), the weighted mean effect was −1.72 kg for fat mass, −0.92% for fat mass percentage, and an increase of 0.63 kg for fat-free mass. In the present study, the reduction was −1.87 kg (−11.78%) for fat mass, −10.07% for fat mass percentage, an increase of 0.35 kg (0.61%) (not significant) for lean mass, and 1.95% for lean mass percentage. The differences observed between the present study and the interventions with HIIT could be due to the great disparity of TID, the duration of the training protocols, and the fact that HIIT is much more stressful and does not allow a large training volume. Therefore, Z1 and Z2 training time may have had a greater effect on reducing fat mass in the present study, and HIIT, while also contributing to fat mass loss, appears to have a greater effect on increasing fat-free mass [24,25].

In fact, HIIT is a training zone generally above 4 mMol·L^−1^ blood lactate, and it has been mentioned that it is an intensity of strong stimulation for improving certain physical parameters and performance [18,26]. These results appear to be quite rapid, but plateau effects also appear to occur prematurely. To avoid premature stagnation and ensure long-term development, training volume should be systematically increased during base training, and the HIIT focus should be more in the build-up phase of the mesocycle training period for recreational cyclists [18].

In the present study, the athletes also showed an increase in the mean percentage of lean mass and body water, perhaps motivated by the reduction in fat mass and increase in plasma volume [27]. Therefore, these somatic changes may be relevant not only for improving cycling performance, but also for promoting the health of recreational cyclists. Indeed, cycling has been associated with several health benefits, namely contributing to the reduction in risk factors for chronic diseases [28], such as lower CVD risk and mortality [29]. However, low bone density has been observed in cyclists, more so in trained athletes with higher weekly training volumes than in recreational athletes [30]. In fact, lower bone mineral density has been observed in endurance athletes, particularly at the level of the lumbar spine [31], compared to wrestlers and judo practitioners, perhaps because these activities have a greater impact on the whole body during daily training compared to subjects in low-impact or non-impact activities [32]. It is important for recreational athletes to understand how cycling may affect their health so that they can practice in a more informed manner.

### 4.3. Power Findings

As for the power variables, P4 showed the greatest improvement between assessment moments. The % P4 presented a significant and high relationship with Z2, and a moderate relationship with Z1, with more time spent in Z2 promoting greater P4 improvement. This relationship has also been observed in amateur Half-Ironman distance triathletes, with training time in zone 2 being associated with improved performance in the Half-Ironman race [15]. Interestingly, this does not seem to be the case with elite athletes, who seem to experience more performance improvements with a polarized approach to TID [26]. In fact, HIIT with short intervals and higher intensity (94 ± 3% of peak aerobic power output—Wmax (%)) appears to induce superior training adaptations compared to long intervals and lower intensity (79 ± 7% of Wmax (%)) in elite cyclists [33]. Nevertheless, the data of the present study show that a significant training volume in the aerobic zones (Z1 and Z2) had a relevant impact on the P4 improvement, possibly mediated by an increase in the lactate clearance capacity of the slow twitch fibers (type I muscle fibers). In addition, training time in these intensity zones can promote significant adaptations in the metabolic pathways of the aerobic system, contributing to an increase in its energy production capacity [27].

There are some important differences between professional and recreational cyclists, namely the fact that professional athletes are already highly aerobically trained, with excellent lactate threshold abilities, and therefore can only really achieve additional gains by spending more time in the Z3. However, less fit non-elite athletes can still achieve good gains from Z2 training. So, while polarized training may be best for elite athletes, the same may not be true for recreational athletes. With this in mind, it is important to remember that what works for professional cyclists may not be ideal for amateur and recreational athletes.

However, the most significant increase was observed in P/W P4. This is partly due to the increase in P4 itself, but also to the significant reduction in body mass, especially fat mass. It has been shown that the higher the intensity of Z1, the greater the fat metabolism, which contributes to the reduction in body mass and consequently to the increase in P/W P4 [34]. These adaptations, associated with the greater ability of the muscles to metabolize fats, are relevant adaptations for improving endurance [27,35,36]. In the study by Alkhatib [34] with 21 healthy men on a cycle ergometer, the maximal fat oxidation was 0.51 ± 0.14 g·min^−1^, corresponding to a blood lactate concentration of 1.4 ± 0.4 mMol·L^−1^, a Fatmax intensity (intensity at which the greatest fat consumption per hour occurs) of 47.2 ± 9.7% ⩒O_2*peak*_ or 40.2 ± 9.4% peak power. However, endurance trained cyclists had their highest fat oxidation (0.67 ± 0.20 g·min^−1^) at 75% ⩒O_2*peak*_ [36]. However, these are intensities performed at Z1.

To determine LaT1 (or aerobic threshold), it is important to note that the 2 mMol·L^−1^ lactate turn point may be difficult to identify in recreational athletes, because blood lactate often approaches this concentration at very low workloads [26]. Therefore, it is very important to start the intensity levels in the aerobic threshold determination protocol with low power values, so that it is possible to reach the intensity threshold corresponding to 2 mMol·L^−1^ of blood lactate concentration in these athletes.

For coaches, this information, along with the existing literature [17], seems to suggest that pyramidal training could be a good strategy for recreational athletes during the base training phase, while polarized training could also be a relevant strategy for the form-building phase and the specialty phase as the day of the competition or event approaches. In this sense, a percentage of the training volume in Z1 and Z2 could have a significant impact on changing somatic variables, namely by reducing the percentage of fat mass, with relevant benefits for improving the P/W P4.

### 4.4. Strengths and Limitations

The most important aspects to highlight from this study are: (i) the high motivation of the participants to diligently follow a structured and planned training program, despite constraints in their personal and professional lives; (ii) the significant improvement in key determinants of sport performance with a training plan that is accessible to recreational cyclists; (iii) that the training plan appears to be suitable for individuals with limited availability for training; (iv) although there may be potentially more suitable training structures, the pyramidal structure used appears to be appropriate for recreational cyclists; and (v) the volume and intensity employed were well tolerated by the cyclists, who reported a perceived recovery from one session to the next, as adapted.

Some limitations can be considered, namely the small sample size, the lack of a control group, the fact that the evaluations were performed in a laboratory environment with a stationary bicycle, the lack of controlled resistance training, and the lack of performance assessment variables. Further research should investigate the intensity levels and training loads performed by recreational cyclists without structured training to understand their patterns, and to compare the effects of pyramidal training and polarized training on the physiological variables that determine performance in these cyclists.

## 5. Conclusions

In this study of recreational and/or recreational cyclists, a TID with a pyramidal approach promoted a significant improvement in P4 and P/W P4 after a 16-week training program. An improvement in somatic variables was also observed, with a decrease in the percentage of fat mass and an increase in the percentage of lean mass. These adaptations may have a relevant impact on their fitness level and performance. Furthermore, training intensities Z1 and Z2, but not Z3, had significant correlations with P4 improvement in these athletes, with Z2 having a more significant effect.

This study suggests that pyramid training may be a good strategy for recreational athletes, especially during the base training phase, to improve P4 and P/W P4, and to promote clinically relevant somatic changes for health promotion. These physiological and somatic adaptations to training are also relevant for the improvement in cycling performance.

## Figures and Tables

**Table 1:** Typical five training zones for prescribing and monitoring training intensity in endurance sports [18].

Intensity Zone	HR_max_ [%max]	Lactate [mMol·L^−1^]
1	55–75	0.8–1.5
2	75–85	1.5–2.5
3	85–90	2.5–4
4	90–95	4–6
5	95–100	6–10

**Table 2:** Total training volume per power zone during the 16-week intervention.

	Mean	SD	CV [%]	Partial Contribution [%]
Workouts [units]	90.14	37.36	41.45	
Distance [km]	3701.36	1181.62	31.92	
TSS [a.u.]	8605.50	4012.67	46.63	
Time [minutes]	9347.14	3151.67	33.72	
Zone 1 [minutes]	4350.80	1673.38	38.46	56.77
Zone 2 [minutes]	2358.20	1132.46	48.02	30.77
Zone 3 [minutes]	954.80	495.81	51.93	12.46

SD—standard deviation; CV—coefficient of variation; TSS—training stress score. Zone 1—blood lactate levels < 2 mMol·L^−1^; Zone 2—blood lactate levels ≥ 2 and <4 mMol·L^−1^; Zone 3—blood lactate levels ≥ 4 mMol·L^−1^ [3].

**Table 3:** Paired-samples *t*-test comparisons with mean difference (MD), 95% confidence intervals (95CI), and the effect size index (Cohen’s d).

	*t*-Test	*p*-Value	MD	95CI	d [Descriptor]
Body mass [kg] ^a^	3.92	<0.001	1.74	0.78 to 2.70	0.19 [trivial]
BMI [kg/m^2^] ^b^	3.64	0.002	0.53	0.21 to 0.84	0.23 [small]
Fat mass [kg]	1.68	0.059	1.87	−0.53 to 4.28	0.40 [small]
Fat mass [%] ^a^	4.07	<0.001	1.99	0.93 to 3.05	0.52 [small]
Lean mass [kg]	−0.89	0.195	−0.35	−1.20 to 0.50	0.06 [trivial]
Lean mass [%] ^a^	−3.86	<0.001	−1.95	−3.04 to −0.86	0.55 [small]
Visceral fat [a.u.] ^b^	3.23	0.003	0.64	1.07 to 3.23	0.26 [small]
H_2_O [%] ^b^	−2.41	0.016	−0.75	−1.41 to −0.08	0.20 [small]
P4 [W] ^a^	−8.21	<0.001	−37.71	−47.64 to −27.79	1.21 [large]
P/W P4 [W/kg] ^a^	−6.79	<0.001	−0.51	−0.34 to −6.79	1.54 [large]
HR P4 [bpm]	−1.00	0.167	−2.36	−7.44 to 2.73	0.20 [small]

BMI—body mass index; H_2_O—water; P4—power at 4 mMol·L^−1^ of blood lactate concentration; P/W P4—power to body mass ratio at P4; HR P4—heart rate at P4. ^a^—significant difference (<0.001) between pre- and post-test; ^b^—significant difference (<0.05) between pre- and post-test.

## Data Availability

Data are unavailable due to privacy or ethical restrictions.
